# Effects of Micellar Quercetin Supplementation on Growth Performance, Nutrient Digestibility, Fecal Microbiota, Meat Quality, and Physiological Status in Broiler Chickens

**DOI:** 10.3390/ani14131918

**Published:** 2024-06-28

**Authors:** Golam Sagir Ahammad, In Ho Kim

**Affiliations:** Department of Animal Biotechnology, Dankook University, No. 29 Anseodong, Cheonan 330-714, Republic of Korea; sagirahbau@gmail.com

**Keywords:** broiler chickens, blood profile, growth performance, quercetin

## Abstract

**Simple Summary:**

The widespread use of antibiotics in poultry nutrition has contributed to antibiotic resistance, negatively impacting poultry health. Consequently, various feed additives have been studied as alternatives to growth promoters to enhance broiler performance. This study investigates the effects of quercetin supplementation on broilers chickens. The results indicate that adding quercetin to the diet improves the broiler performance and meat quality and positively impacts the blood profile parameters. These findings are valuable for developing new feed additives in the broiler industry.

**Abstract:**

This study investigated the impacts of micellar quercetin (MQ) supplementation on growth performance, meat stability, excreta gas emissions, and physiological status. During a 35-day trial, 640 Ross 308 broilers were utilized. These birds were one day old, with an average initial body weight of 43.34 ± 1.43 g. They were randomly distributed across four experimental diets, each consisting of 10 replicate pens with 16 chicks per pen. The diets included the following: control (CON) with 0% micellar quercetin (MQ), TRT1 with 0.025% MQ, TRT2 with 0.050% MQ, and TRT3 with 0.100% MQ. The results indicate that broilers fed diets with increasing levels of MQ exhibited significantly higher body weight gains (BWGs) compared to the control group (*p* < 0.05). There was a clear linear increase in the breast muscle percentage with higher levels of quercetin supplementation (*p* < 0.05), while the breast color remained consistent across all groups (*p* > 0.05). Both cooking loss and drip loss exhibited a linear decrease as MQ levels in the diet increased (*p* < 0.05). The level of aspartate aminotransferase (AST) tended to decrease with higher MQ levels. Thyroxine (T4) and lymphocyte levels also showed a linear increase with increasing MQ dosage in the diet (*p* < 0.05). However, no significant effects were observed on nutrient digestibility, gas emissions, or fecal microbial components (*Lactobacillus*, *E. coli*, and *Salmonella*) with higher levels of MQ supplementation (*p* > 0.05). In conclusion, augmenting quercetin levels in the diet positively influenced the BWG, breast muscle development, and meat quality parameters such as cooking loss and drip loss, with beneficial effects on blood profiles.

## 1. Introduction

The modern broiler industry faces ongoing challenges related to sustainable production, disease resistance, and meat quality [1]. Therefore, achieving optimal growth performance, ensuring meat quality, and bolstering immunological responses are crucial for sustainable and efficient farming practices. However, the presence of diverse viruses, bacteria, parasites, microbial toxins, and nutrient deficiencies can give rise to the development of immunosuppressive diseases [2]. To prevent such diseases, it is crucial to maintain optimal health indicators such as liver function, muscle health, thyroid activity, immune response, and overall stress levels [3]. Therefore, the quest for natural and effective feed additives has intensified. As a result, quercetin, a bioactive flavonoid abundant in various plant sources, has emerged as a promising candidate with potential benefits for broiler chickens [4]. Quercetin is a prominent dietary flavonoid, widely found in vegetables, fruits, tea, and wine [5]. Additionally, quercetin offers health benefits by positively influencing metabolic processes, regulating inflammation, and reducing serum cholesterol levels [5,6]. Quercetin’s stable chemical structure and water-soluble derivatives enable effective in vivo absorption, making it a versatile additive for various applications in production practices [7]. Earlier studies indicate that quercetin supplementation exhibits numerous pharmacological activities, such as antioxidative, anti-inflammatory, and antibacterial properties, as well as the ability to scavenge free radicals and enhance immune functions [8]. For example, adding quercetin at a concentration of 1 g/kg to broiler diets can extend the shelf life of meat by slowing down lipid oxidation [9]. Furthermore, supplementing broiler chick diets with MQ (0.2, 0.4, or 0.6 g/kg) improved breast muscle yield and quality, enhanced dry matter digestibility, and increased energy retention. These effects were attributed to elevated counts of beneficial bacteria in the ceca, leading to enhanced growth performance [10]. Despite the positive impacts of flavonoids like quercetin on antioxidant and immune systems, their effects on growth performance remain inconsistent [11,12]. Additionally, high levels of quercetin can negatively affect feed conversion ratios [9]. Due to its low solubility, researchers are investigating micellar quercetin, which offers a higher solubility. However, studies on the effects of micelle-type quercetin on the growth performance, meat quality, nutrient digestibility, cecal microbiota, gas emissions, and blood profiles are still limited.

Based on the above discussion, we hypothesize that supplementing broiler diets with MQ will enhance the growth performance and improve meat quality by positively affecting physiological parameters in broiler chickens. The objective of the present study was to assess the effects of MQ supplementation in the diet on the growth performance, nutrient digestibility, meat quality, cecal microbial population, excreta gas emissions, and blood profile in broilers.

## 2. Materials and Methods

### 2.1. Animals and Experimental Design

In a 35-day trial, 640 one-day-old Ross 308 broilers (male) weighing 43.34 ± 1.43 g were used. The broilers were randomly allotted to 4 experimental diets with 10 replicates of 16 chicks per pen. The dietary treatments consisted of CON, a basal diet with 0% MQ; TRT1, a basal diet with 0.025% MQ; TRT2, a basal diet with 0.050% MQ; and TRT3, a basal diet with 0.100% MQ. The quercetin used in our study, which was 97% pure, was sourced from Synergen (190, Sinheung, Bucheon, Gyeonggi, South Korea). The diets were formulated according to the guidelines provided by the NRC [13] (Table 1). The broilers were raised in a three-tiered stainless-steel battery pen (1.75 long × 1.55 wide × 0.5 high, m), with ad libitum access to water and feed. The entire housing facility was environmentally controlled throughout the experiment. The broilers were equally distributed between the upper and lower pens to minimize the effect of the pen level. For the first 10 days, each pen was equipped with two plastic feeders containing 1.2 kg of feed each. Subsequently, a feed trough measuring 1.75 m in length was installed on both the front and back sides of the pen. During the initial week, the temperature in the broiler house was maintained at 33 ± 1 °C. After the first week, the temperature was gradually reduced to 24 ± 1 °C, ensuring that the relative humidity remained above 60%.

### 2.2. Growth Performance

Body weight measurements were taken on days 7, 21, and 35. To evaluate the BWGs in broilers, each bird was weighed at these time points, and the difference from their initial weight was calculated to determine the weight gained. The feed intake (FI) was measured by subtracting the amount of remaining feed from the initial amount at each weighing session. The feed conversion ratio (FCR) was then calculated using the FI and BWG values obtained.

### 2.3. Nutrient Digestibility

During the final 7 days of the experiment, feed supplemented with chromium oxide (Cr_2_O_3_, 0.20%) was utilized to evaluate the nutrient digestibility [14]. After 35 days, excreta samples were collected, dried at 60 °C for 72 h, and then pulverized for analysis. The feed mixed with Cr_2_O_3_ was analyzed for its gross energy (GE), dry matter (DM), and nitrogen (N) content using the AOAC (2000) method. Ultraviolet (UV) absorption spectrophotometry (UV-1201, Shimadzu, and Kyoto, Japan) was employed to determine the chromium levels. Digestibility was calculated using the following formula: Digestibility = 1 − [(*N_f_* × *C_d_*)/(*N_d_* × *C_f_*)] × 100, where *N_f_* = the concentration of nutrient in the excreta (% DM), *N_d_* represents the concentration of the nutrient in the diet, *C_d_* represents the concentration of chromium in the diet, and *C_f_* represents the concentration of chromium in the excreta.

### 2.4. Meat Quality and Organ Weight

After the 35-day trial, a total of 120 broilers (3 broilers per replication) were slaughtered using cervical dislocation following bleeding from the jugular vein. Skilled personnel then removed and weighed the liver, spleen, bursa of Fabricius, breast meat, abdominal fat, and gizzard from each bird. The weights of these organs were then expressed as a percentage of body weight. After slaughter, the sections were individually packed in plastic bags and stored at 4 °C for 24 h for pH analysis. The pH of the meat samples was measured three times using a glass electrode pH meter (WTW pH 340-A, WTH Measurement Systems Inc., Fort Myers, FL, USA) with a penetration glass electrode. The pH was measured 24 h post-mortem.

At 24 h following slaughter, the color characteristics of the breast meat were assessed using a Minolta CR410 colorimeter (Konica Minolta Sensing Inc., Osaka, Japan), which was calibrated using a standard white plate with values of L* = 93.5, a* = 0.3132, and b* = 0.3198. The chromameter was powered on and calibrated, then its sensor head was positioned directly on the meat. For accuracy, four time readings were taken from different areas, and the results were averaged.

The water-holding capacity (WHC) was measured following the procedure described in [15]. Briefly, a 0.3 g sample was pressed at 3000× *g* for 3 min at 26 °C on a 125 mm-diameter piece of filter paper. The areas of the compressed sample and the expelled moisture were outlined and subsequently measured using a digital area line sensor (MT-10S, M.T. Precision Co., Ltd., Tokyo, Japan). The water-to-meat area ratio was then calculated to determine the WHC. To assess cooking loss, 4 g meat samples were placed in plastic containers, submerged in a 75 °C water bath for 30 min, cooled, and weighed, and the weight difference was calculated as a percentage [16]. For drip loss assessment, 4 g meat samples were stored in plastic bags at 4 °C [16]. The weights of these samples were recorded on days 1, 3, 5, and 7, and the percentage of weight reduction was calculated.

### 2.5. Cecum Microbiota

Excreta samples were collected from the ceca of birds housed in 10 pens per treatment group. These samples were kept chilled during transportation (40 min) to ensure their integrity. Upon arrival at the study site, the chilled samples were promptly subjected to microbiological examination for detailed analysis. The chilling during transportation was crucial to preserve the samples in their original state, preventing any microbial growth or changes that could affect the accuracy of the analysis. Once at the laboratory, the samples were carefully analyzed to determine their microbiological composition. First, 1 g of each sample it was mixed with 9 mL of sterile peptone broth, then the mixture was shaken for 60 s. Next, 10-fold serial dilutions of the mixture were made, ranging from a 1:10 dilution to a 1:100,000,000 dilution. These dilutions were plated on three different types of agars: *Lactobacilli* MRS agar for lactic acid bacteria, MacConkey agar for *E. coli*, and *Salmonella*–*Shigella* agar for *Salmonella.* The *Lactobacilli* MRS agar plates were incubated without oxygen (anaerobically) at 37 °C for 24 h, and the MacConkey and *Salmonella*–*Shigella* agar plates were incubated with oxygen (aerobically) at 37 °C for 24 h. After incubation, the bacterial colonies on each type of agar were counted. Finally, the results were reported as the logarithm of colony-forming units per gram (log10 CFU/g) to indicate the number of bacteria present in each gram of the original sample.

### 2.6. Excreta Gas Emission

During the final week of the study, fresh excreta samples (300 g each) were collected from every replication pen over a span of four consecutive days. These samples were gathered to evaluate the emissions of ammonia (NH_3_), hydrogen sulfide (H_2_S), total mercaptans, carbon dioxide (CO_2_), and acetic acid. For the purpose of analysis, the excreta samples were placed in 2.6 L sealed plastic containers, with two containers designated for each sample. These containers were then stored for one week at a consistent room temperature of 25 °C. As fermentation progressed, the levels of NH_3_, H_2_S, total methyl mercaptans, CO_2_, and acetic acid in the samples were measured using a MultiRAE Lite model PGM-6208 gas meter (RAE Systems, Raleigh, NC, USA).

### 2.7. Blood Profile

At the end of the trial, 5 mL of blood was taken from 120 (3 broilers per replicate) broilers using the wing vein puncher. These samples were deposited in K2-EDTA heparinized tubes (K2-EDTA, BD Vacutainer, Plymouth, Devon, UK). For whole blood retrieval, tubes equipped with K3-EDTA from Becton Dickison Vacutainer systems were employed. After collecting the samples, they were spun in a centrifuge at 3000 rotations per minute for 15 min to separate the plasma from the blood, which was then transferred to small Eppendorf tubes and stored in a freezer at −20 °C until analysis. The concentrations of aspartate aminotransferase (AST), alanine aminotransferase (ALT), lactate dehydrogenase (LDH), creatine kinase, triiodothyronine (T3), thyroxine (T4), lymphocyte, hematocrit, heterophil, and HSP70 were determined using an automatic blood biochemistry analyzer (Konelab 20 analyzer, Thermo Fisher Scientific, Vantaa, Finland), using commercial diagnostic kits (Quantitation Kit; Bethyl Laboratories, Montgomery, TX, USA) and following the manufacturer’s guidelines.

### 2.8. Statistical Analysis

The data analysis was conducted using the General Linear Model (GLM) procedure within SAS (SAS Inst. Inc., Carry, NC, USA). We assessed the linear and quadratic effects of increasing dietary quercetin supplementation through polynomial orthogonal contrasts. Each replicate was treated as an experimental unit for analysis. Data variability was quantified using the standard error of the mean (SEM). Statistical significance was declared for *p*-values below 0.05, with trends recognized at a *p*-value threshold below 0.10.

## 3. Results

### 3.1. Growth Performance

The effect of quercetin on the growth performance is presented in Table 2. Overall, the BWG increased linearly with the increasing inclusion of supplemental quercetin (*p* < 0.05). However, the feed intake (FI) and feed conversion ratio (FCR) were unchanged with the increasing inclusion of quercetin in the broiler diets (*p* > 0.05).

### 3.2. Nutrient Digestibility

No difference in the nutrient digestibility (dry matter, nitrogen, and digestible energy) was found between the treatments (*p* > 0.05) (Table 3).

### 3.3. Meat Quality and Organ Weights

The effects of quercetin on the meat quality and organ weights are shown in Table 4. The increasing inclusion of quercetin supplementation linearly reduced the cooking loss and drip loss on day 3 while increasing the breast muscle (*p* < 0.05). However, the pH values, breast muscle color, and relative organ weights (liver, Bursa of Fabricius, abdominal fat, spleen, and gizzard) did not show significant changes with the increasing inclusion of quercetin in broiler diets (*p* > 0.05).

### 3.4. Cecum Microbiota

The data on the cecum microbiota (*Lactobacillus*, *E. coli*, and *Salmonella)* is displayed in Table 5. No significant difference in the cecum microbes was found (*p* > 0.05).

### 3.5. Gas Emissions

Table 6 presents the data on excreta gas emissions (NH_3_, H_2_S, methyl mercaptans, CO_2_, and acetic acid), indicating that no significant differences were observed (*p* > 0.05).

### 3.6. Blood Profile

The effect of quercetin supplementation on the blood profile of broilers is shown in Table 7. The level of AST tended to reduce with increasing levels of quercetin (*p* < 0.1). T4 and lymphocytes also showed a linear increase with a higher quercetin dosage in the diet (*p* < 0.05). No significant differences were found among the treatments for other blood parameters (*p* > 0.05).

## 4. Discussion

Our current study aligns with the findings of [17], which demonstrated that incorporating soybean isoflavones (at levels ranging from 10 to 80 mg/kg) in chicken diets increased the BWGs in broilers. Similarly, a diet containing 0.050% quercetin significantly improved the BWGs in broilers [11]. Additionally, including 2 mg/kg of kaempferol in the diet significantly increased the BWGs, FIs, and FCRs in broilers [18]. However, while thymol (200 mg/kg of feed) had no impact on the broiler growth parameters, carvacrol at the same level decreased the growth rate and feed intake but improved the feed conversion ratio [19]. Moreover, cinnamaldehyde (200 mg/kg of feed) had no effect on the growth performance of female broiler chickens [12]. Likewise, quercetin supplementation at concentrations of 0.5 and 1 g/kg of feed did not significantly alter the BWG or FI [9]. Again, the inclusion of 5 mg of genistein/kg of feed and 20 mg of hesperidin/kg of feed had no impact on the BWG, FI, and FCR [9]. Similarly, hesperidin dietary supplementation, up to 3 g/kg of feed, had no effect on the BWGs, FIs, and FCRs of broilers [20]. Furthermore, feeding silymarin at 40 and 80 ppm had no effect on the growth performance of broilers [21]. The differing results may be attributed to the different types and doses of flavonoid substances in broiler diets. High doses of quercetin might negatively influence the growth performance due to its poor solubility and absorbability [9]. However, micelle-type quercetin increases the solubility and absorbability of quercetin [22]. Our study indicates that incorporating micelle-form quercetin at low levels enhances its absorbability, potentially increasing broiler BWGs by improving flavonoid absorption.

Adding 200 mg/kg of flavonoids from *Scutellaria baicalensis* improved the nutrient digestibility in broiler chickens [23]. Moreover, a dietary addition of 0.050% quercetin flavonoids enhanced the nutrient digestibility in broiler chickens [11]. Similarly, administering 0.06% quercetin as supplementation demonstrated a linear enhancement in both the dry matter and energy digestibility [15]. Furthermore, incorporating 6.4 mg/kg of quercetin into broiler diets improved the apparent digestibility of crude fat and crude protein [24]. Likewise, supplementing broiler chicken diets with a lacquer-containing flavonoid complex at 1%, 2%, and 4% improved the digestibility of crude protein and fat compared to the control diet [25]. However, our study aligns with the findings reported in [26], which indicated no change in the nutrient digestibility in rabbits given 2 g/kg quercetin as supplementation and no alteration in the cecum microbial count. It is well known that the microbiota plays a crucial role in nutrient digestion. Our study found no significant effect on the nutrient digestibility, likely due to an unchanged microbial composition.

Carcass yields remain economically significant for producers, while sensory attributes like color, flavor, and texture tend to be pivotal factors influencing consumer purchasing decisions. Our study found that quercetin supplementation did not significantly affect the pH value, breast muscle color (lightness, redness, or yellowness), or water-holding capacity (WHC). However, it did significantly impact the breast meat yield, cooking loss, and drip loss. There were no differences in the meat color or ultimate pH in broilers fed a diet supplemented with 0.75 g/kg of hesperidin [27]. Moreover, broilers fed a diet supplemented with 1.5 g/kg of hesperidin showed no differences in their meat color or ultimate pH [20]. Likewise, supplementation with a fermented citrus product containing quercetin and rutin at levels of 0.25–1 g/kg feed [28], as well as a propolis extract at levels of 0.5–3 g/kg feed [29], showed similar effects to those observed in our study. However, adding 40 mg/kg of isoflavones to the feed caused the breast fillets of male broiler chickens to have a lighter color (indicating higher L* values) and higher pH values [17]. This implies that the observed outcomes could vary depending on the specific dosages and sources of supplementation utilized. Our study showed that quercetin supplementation in the diet reduced the cooking loss and drip loss linearly. Cooking loss is influenced by oxidative stress, which causes protein denaturation and moisture loss during cooking, ultimately leading to an increased cooking loss [27]. Quercetin scavenges free radicals and inhibits lipid peroxidation, protecting proteins from oxidative damage and preserving their structure and functionality [30]. Additionally, quercetin may influence the muscle metabolism, collagen content, and water-binding capacity [31,32], all of which can impact cooking and drip losses. This mechanism may contribute to a decreased cooking loss in broilers. Our study is consistent with [33], which reported significant changes in the percentages of breast muscle with kaempferol concentrations of 0.3% or 0.6%, without altering the weights of other organs. Similarly, incorporating flavonoids from oregano, cinnamon, and pepper into the diet did not produce any significant impact on the weights of the gizzard, liver, pancreas, large intestine, or small intestine [34]. Moreover, 0.06% of quercetin supplementation linearly improved the relative organ weights of the breast muscle [15]. Quercetin’s specific interactions with molecular pathways in muscle tissues may influence muscle development, protein synthesis, or myogenesis, potentially leading to increased breast muscle weights [35].

Our study aligned with [36,37], which found no significant effects of phytogenic supplementation with essential oils and flavonoids on the *E. coli* and *Lactobacillus* populations in broiler ceca. Similar results were observed with 0.050% quercetin supplementation [11]. Moreover, there was no statistical impact on intestinal microbial numbers with the supplementation of 250 mg/kg and 500 mg/kg of quercetin [38]. In contrast, supplementing with onion skin extract reduced the levels of *Lactobacillus*, *E. coli*, and *Salmonella* in the ceca of broiler chickens [39]. Additionally, fermented plant extracts rich in flavonoids reduced the *E. coli* levels without impacting the *Lactobacillus* or *Salmonella* populations in broilers, suggesting that this antimicrobial action could be attributed to the presence of lactic acid and organic acids in the fermented flavonoid source, which they described as a synergistic effect of flavonoids and organic acids [40]. These findings imply that among the flavonoids studied, only quercetin lacked antimicrobial efficacy against intestinal bacteria.

Our current study stated that there was no difference between the groups in terms of the excreta NH_3_, H_2_S, and methyl mercaptans emissions with quercetin supplementation in the diet. Similarly, there is no significant effect on the gas emissions or nutrient digestibility with 0.05% quercetin supplementation [11]. Moreover, changes in the nutrient digestibility would bring about changes in the gas emissions in growing pigs [41]. Quercetin did not influence any digestibility parameters, so no difference in the excreta gas emissions is also logical.

The present study showed that an increasing level of supplemented quercetin had a tendency for AST and a significant effect on T4 and lymphocytes in broilers. The better condition of the liver indicates that the AST level decreased in the serum of the broiler. Conversely, liver damage occurred, accompanied by an increase in the AST levels in the blood serum of a broiler [42]. Moreover, liver damage coincided with increased oxidative stress and free radical levels, leading to elevated AST levels. The decrease in AST levels indicates a possible beneficial effect of flavonoids on liver health. Flavonoids possess hepatoprotective properties, effectively neutralizing free radicals and alleviating oxidative stress in the body [30]. Our study corroborates the findings of [43], which showed that flavonoids of *Rhizoma drynariae* reduced the AST levels in the broiler. Thyroxine plays a crucial role in various physiological functions essential for the health, growth, and productivity of broilers. Iodine and selenium are essential for thyroxine hormone synthesis [44]. Flavonoids may improve the uptake of iodine by thyroid follicular cells, which is essential for thyroid hormone synthesis [45]. Flavonoids may regulate the activity of thyroid peroxidase, an enzyme involved in the synthesis of thyroid hormones [46]. The previous study showed that 0.05% quercetin supplementation increased the T4 hormone in the blood of broilers [47]. Lymphocytes play a role in forming antibodies that circulate in the blood and developing the cellular immune system [48]. Stress in broilers increases the secretion of glucocorticoid hormones, which in turn decreases lymphocyte levels in the blood [49]. Flavonoids have been shown to enhance immune responses by safeguarding against pro-inflammatory cytokines, regulating the proliferation of immune cells, and mitigating stress through various mechanisms [50]. Supplementation with 1 g/kg of quercetin enhanced the immune parameters in broilers, which aligns with our results [51].

## 5. Conclusions

In conclusion, this study demonstrates that incorporating MQ into broiler diets at levels up to 0.100% can significantly enhance the growth performance and meat quality without negatively impacting the nutrient digestibility, cecal microbial composition, or gas emissions. Furthermore, MQ supplementation may confer health benefits by improving the T4 and lymphocyte levels. These findings suggest that MQ supplementation at levels up to 0.100% could be advantageous for the broiler industry.

## Figures and Tables

**Table 1:** Ingredient compositions of experimental diets on as-fed basis.

Ingredient, %	Starter	Grower	Finisher
Corn	54.19	55.38	56.77
Soybean meal	33.80	26.1	18.23
Canola meal	5.00	10.0	15.0
Soybean oil	2.10	3.62	5.07
MDCP ^1^	-	1.28	1.12
DCP ^2^	1.70	-	-
Limestone	1.15	1.34	1.22
L-lysine	0.50	0.65	0.81
DL-methionine	0.46	0.47	0.52
L-threonine	0.20	0.25	0.32
L-tryptophan	-	0.01	0.04
NaHCO_3_	0.10	0.10	0.10
Salt	0.30	0.30	0.30
Vitamin premix ^3^	0.20	0.20	0.20
Mineral premix ^4^	0.20	0.20	0.20
Choline	0.10	0.10	0.10
Nutrient composition			
Metabolic energy, kcal/kg	3000	3100	3200
Crude protein, %	23.0	21.5	20.0
Lysine, %	1.50	1.40	1.30
Methionine + cysteine, %	1.08	0.99	0.94
Available phosphorus, %	0.48	0.44	0.41
Calcium, %	0.96	0.87	0.81

^1^ Monodicalcium phosphate. ^2^ Dicalcium phosphate. ^3^ Provided per kg of complete diet: 11,025 IU vitamin A; 1103 IU vitamin D_3_; 44 IU vitamin E; 4.4 mg vitamin K; 8.3 mg riboflavin; 50 mg niacin; 4 mg thiamine; 29 mg d-pantothenic; 166 mg choline; 33 μg vitamin B_12_. ^4^ Provided per kg of complete diet: 12 mg Cu (as CuSO_4_·5H_2_O); 85 mg Zn (asZnSO_4_); 8 mg Mn (as MnO_2_); 0.28 mg I (as KI); 0.15 mg Se (as Na_2_SeO_3_·5H_2_O).

**Table 2:** The effect of micellar quercetin supplementation in the diet on growth performance in broilers ^1^.

Items	CON	TRT1	TRT2	TRT3	SEM ^2^	*p*-Value	
						Linear	Quadratic
d 1 to 7							
BWG, g	114 ^b^	115 ^ab^	116 ^ab^	123 ^a^	2.89	0.0389	0.3862
FI, g	136	140	137	144	2.53	0.0748	0.4367
FCR	1.204	1.215	1.2	1.178	0.03	0.5524	0.6292
d 7 to 21							
BWG, g	725	733	735	748	6.83	0.0264	0.7175
FI, g	1002	1011	1019	1028	10.44	0.0824	0.9735
FCR	1.383	1.381	1.388	1.375	0.01	0.8313	0.7776
d 21 to 35							
BWG, g	923 ^b^	935 ^ab^	972 ^a^	987 ^a^	19.51	0.0146	0.9272
FI, g	1720	1747	1753	1759	34.23	0.4365	0.7603
FCR	1.867	1.875	1.811	1.789	0.04	0.1376	0.7324
Overall							
BWG, g	1761 ^b^	1783 ^b^	1822 ^ab^	1857 ^a^	18.06	0.0004	0.6994
FI, g	2859	2898	2908	2931	37.71	0.1901	0.8265
FCR	1.624	1.626	1.597	1.579	0.22	0.1117	0.6421

^1^ Abbreviation: CON, basal diet; TRT1, CON + 0.025% micellar quercetin; TRT2, CON + 0.050% micellar quercetin; TRT3, CON + 0.100% micellar quercetin; BWG, body weight gain; FI, feed intake; FCR, feed conversion ratio. ^2^ Standard error of means. Means in the same row with different superscripts differ significantly (*p* < 0.05).

**Table 3:** The effect of micelle quercetin supplementation in the diet on nutrient digestibility in broilers ^1^.

Items, %	CON	TRT1	TRT2	TRT3	SEM ^2^	*p*-Value	
						Linear	Quadratic
D35							
Dry matter	70.76	71.77	72.13	72.20	1.45	0.5871	0.9493
Nitrogen	68.32	69.36	69.91	70.03	1.44	0.5404	0.9457
Digestible energy	70.10	71.71	71.96	71.82	1.66	0.5513	0.7882

^1^ Abbreviation: CON, basal diet; TRT1, CON + 0.025% micellar quercetin; TRT2, CON + 0.050% micellar quercetin; TRT3, CON + 0.100% micellar quercetin. ^2^ Standard error of means.

**Table 4:** The effects of micelle quercetin supplementation in the diet on the meat quality and organ weights in broilers ^1^.

Items	CON	TRT1	TRT2	TRT3	SEM ^2^	*p*-Value	
						Linear	Quadratic
pH value	5.40	5.43	5.48	5.50	0.06	0.5109	0.6891
Breast muscle color							
Lightness (L*)	59.76	57.73	60.54	59.36	1.30	0.7869	0.7483
Redness (a*)	11.28	10.73	10.17	10.45	0.62	0.3223	0.5408
Yellowness (b*)	11.65	10.56	11.26	11.33	0.68	0.9356	0.4208
WHC, %	50.09	51.18	51.39	51.63	4.45	0.8131	0.9258
Cooking loss, %	18.40 ^a^	16.43 ^ab^	15.86 ^ab^	14.98 ^b^	1.17	0.0422	0.6518
Drip loss, %							
d 1	3.42	3.53	3.34	3.22	0.24	0.4660	0.6400
d 3	7.57 ^a^	6.78 ^ab^	6.77 ^ab^	5.76 ^b^	0.49	0.0305	0.8312
d 5	14.51	14.40	14.35	13.79	0.63	0.4556	0.7297
d 7	23.32	23.76	22.79	22.81	0.91	0.5530	0.8217
Breast muscle %	16.49 ^b^	17.03 ^ab^	17.44 ^ab^	18.04 ^a^	0.51	0.0497	0.9503
Relative organ weight, %							
Liver	2.68	2.60	2.65	2.67	0.22	0.9970	0.8260
Bursa of Fabricius	0.13	0.12	0.14	0.14	0.02	0.4895	0.7729
Abdominal fat	0.82	0.74	0.72	0.81	0.10	0.9207	0.4545
Spleen	0.12	0.13	0.14	0.14	0.02	0.2260	0.8082
Gizzard	1.69	1.59	1.71	1.83	0.16	0.5070	0.5236

^1^ Abbreviation: CON, basal diet; TRT1, CON + 0.025% micellar quercetin; TRT2, CON + 0.050% micellar quercetin; TRT3, CON + 0.100% micellar quercetin. ^2^ Standard error of means. Means in the same row with different superscripts differ significantly (*p* < 0.05).

**Table 5:** The effect of micelle quercetin supplementation in the diet on the microbiota in broilers ^1^.

Items, log_10_ cfu/g	CON	TRT1	TRT2	TRT3	SEM ^2^	*p*-Value	
						Linear	Quadratic
D 35							
*Lactobacillus*	8.86	8.93	8.96	8.93	0.08	0.5152	0.5472
*E. coli*	6.03	5.69	5.83	5.95	0.11	0.8234	0.0529
*Salmonella*	4.42	4.30	4.39	4.29	0.09	0.4936	0.9237

^1^ Abbreviation: CON, basal diet; TRT1, CON + 0.025% micellar quercetin; TRT2, CON + 0.050% micellar quercetin; TRT3, CON + 0.100% micellar quercetin. ^2^ Standard error of means.

**Table 6:** The effect of micelle quercetin supplementation in the diet on gas emissions in broilers ^1^.

Items, ppm	CON	TRT1	TRT2	TRT3	SEM ^2^	*p*-Value	
						Linear	Quadratic
D 35							
NH_3_	17.0	12.8	12.5	12.8	2.63	0.6154	0.2304
H_2_S	2.6	2.1	2.1	2.2	0.46	0.3039	0.8936
Methyl mercaptans	6.5	6.3	7.3	5.8	1.57	0.8612	0.5853
CO_2_	1850	1600	1550	1525	320.17	0.7175	0.6171
Acetic acid	3.5	2.5	4.0	2.8	0.88	0.7552	0.3339

^1^ Abbreviation: CON, basal diet; TRT1, CON + 0.025% micellar quercetin; TRT2, CON + 0.050% micellar quercetin; TRT3, CON + 0.100% micellar quercetin. ^2^ Standard error of means.

**Table 7:** The effect of micelle quercetin supplementation in the diet on the blood profile in broilers ^1^.

Items	CON	TRT1	TRT2	TRT3	SEM ^2^	*p*-Value	
						Linear	Quadratic
D35							
AST, U/L	305.8	270.0	255.8	249.8	22.05	0.0976	0.5169
ALT, U/L	4.8	4.3	4.0	3.3	0.62	0.1235	0.8459
Lactate dehydrogenase, U/L	4055.5	3019.3	3349.8	2986.3	465.37	0.2002	0.4882
Creatine kinase, U/L	7084.3	4275.5	6953.5	4209.8	1748.91	0.4666	0.9856
T3, ng/dL	167.5	144.0	133.8	142.5	15.25	0.2429	0.3180
T4, ug/dL	1.01 ^b^	1.19 ^ab^	1.34 ^ab^	1.57 ^a^	0.18	0.0500	0.9048
Lymphocyte, %	73.60 ^b^	76.58 ^ab^	78.48 ^ab^	80.56 ^a^	11.40	0.0408	0.3152
Hematocrit, %	36.25	34.35	34.93	36.20	1.20	0.9390	0.2213
Heterophil, %	8.10	7.70	7.75	7.90	0.37	0.7482	0.4780
HSP70, ng/mL	0.40	0.31	0.03	0.06	0.14	0.0712	0.6828

^1^ Abbreviation: CON, basal diet; TRT1, CON + 0.025% micellar quercetin; TRT2, CON + 0.050% micellar quercetin; TRT3, CON + 0.100% micellar quercetin; AST, aspartate aminotransferase; ALT alanine aminotransferase; LDH, lactate dehydrogenase; T3, triiodothyronine; T4, thyroxine. ^2^ Standard error of means. Means in the same row with different superscripts differ significantly (*p* < 0.05).

## Data Availability

The data presented in this study can be obtained by requesting them from the corresponding author. They are not publicly available due to ethical restrictions.
